# Supplementary material for: Integration of Micro-Nano-Engineered Hydroxyapatite/Biochars with Optimized Sorption for Heavy Metals and Pharmaceuticals
Source: Nanomaterials (Basel). 2022 Jun 9;12(12):1988. doi: 10.3390/nano12121988 (PMC9227354; doi:10.3390/nano12121988)
Supplement: Supplementary file 1 [file nanomaterials-12-01988-s001.zip › nanomaterials-1724735-supplementary.pdf]

nanomaterials

Supplementary Materials

Integration of Micro-Nano-Engineered Hydroxyapatite/Biochars with Optimized Sorption for Heavy Metals and Pharmaceuticals

Xin Zhao [1], Peiling Yuan [2,*], Ziyan Yang [3,4], Wei Peng [5], Xiang Meng [6] and Jiang Cheng [6]

[1] Graduate Department, Civil Aviation Flight University of China, Guanghan 618307, China; zhaox@cafuc.edu.cn
[2] College of Science, Zhengzhou Key Laboratory of Low-dimensional Quantum Materials and Devices, Zhongyuan University of Technology, Zhengzhou 450007, China
[3] School of Environmental and Municipal Engineering, North China University of Water Resources and Electric Power, Zhengzhou 450045, China; yangziyan@ncwu.edu.cn
[4] Henan Province Key Laboratory of Water Pollution Control and Rehabilitation Technology, Henan University of Urban Construction, Pingdingshan 467036, China
[5] Department of Ecology and Environment of Henan Province, Zhengzhou 450046, China; weipeng_henan@outlook.com
[6] Chongqing Key Laboratory of Materials Surface & Interface Science, Chongqing University of Arts and Sciences, Chongqing 402160, China; xmeng@cqwu.edu.cn (X.M.); cheng20120027@hotmail.com (J.C.)
* Correspondence: yuan.peiling.18@zut.edu.cn

1. Batch Experiments

1.1. Sorption of Heavy Metals

The stock solutions (500.0 mg/L) of Pb(II), Cu(II), and Cd(II) were prepared by dissolving $Pb(NO_3)_2$, $Cu(NO_3)_2$, and $Cd(NO_3)_2$ in DI water, respectively. All the batch sorption experiments were performed in conical flasks located on an oscillating box at a frequency of 200 rpm for 24 h. The stock suspension of HBCs, heavy metals, and $NaNO_3$ solution were mixed to achieve the desired concentrations of each component. Specifically, the effects of geochemical conditions, including contact time (0.0–1440.0 min), pH value (2.0–7.0), ionic strength (0.01–0.20 mol/L), coexisting HA molecule (0–30 mg/L), and temperature (293–313 K), were studied, respectively. After being shaken for 24 h (enough to achieve the sorption equilibrium in this study), the solid/liquid phases in suspensions were separated by centrifuging at 14,000 rpm for 10 min, and the obtained supernatants were filtrated with a 0.22 mm filter membrane. The concentrations of Pb(II), Cu(II), and Cd(II) were determined by flame atomic absorption spectrometry (AA-6880, Shimadzu, Kyoto, Japan). The sorption capacity (q_e, mg/g) of heavy metals was calculated from the initial (C_0, mg/L) and final equilibrium concentration (C_e, mg/L) as $q_e=(C_0–C_e)/(m/V)$, where m/V (g/L) was the dosage of sorbents. All experimental data were the averages of triplicate determinations, and the relative errors of the data were less than 5.0%.

The compositions of simulated wastewater referred to previous reports as follows: 4.0 mg/L of $CaNO_3·2H_2O$, 7.0 mg/L of $NaNO_3$, 180.0 mg/L of NH_4NO_3, 40.0 mg/L of $K_2HPO_4·3H_2O$, 205.0 mg/L of CH_3COONa, 3.4 mg/L of $MgCl_2·6H_2O$, and 110.0 mg/L of C_6H_5COONa. $Cu(NO_3)_2$, $Cd(NO_3)_2$, and $Pb(NO_3)_2$ were added to the artificial wastewater with an initial concentration of 60.0 mg/L for each heavy metal. The solution pH was adjusted to 2.0 and 4.0, respectively. A total of 0.05 g of HBCs was mixed with 100.0 mL of artificial wastewater at 293 K with an oscillation of 24 h. Then, the suspension was centrifuged and filtered through the nylon membranes with a pore size of 0.22 μm. The concentrations of Cu(II), Cd(II), and Pb(II) were determined by flame atomic absorption spectroscopy.

1.2. Sorption of Pharmaceuticals

The sorption kinetics of CBZ and TC on HBCs were studied by adding HBCs (500.0 mg) into pharmaceutical solutions (100.0 mg/L of CBZ and TC separately, 1000.0 mL) with constant stirring at 293 K. The investigation of sorption isotherms of CBZ and tetracycline were carried out in conical flasks with C_0 from 10 to 100 mg/L. The effects of geochemical conditions, including contact time (0.0–1440.0 min), pH value (2.0–8.0), ionic strength (0.01–0.20 mol/L), and temperature (293–313 K), were studied. The suspensions with given time intervals were centrifuged and filtered through a 0.22 mm nylon membrane. The concentrations of CBZ and TC in supernatants were measured by a UV spectrophotometer (Shimadzu 2600) at the maximum absorbance wavelength of 284 and 276 nm, respectively.

1.3. Regeneration Studies

The regenerations of HBCs after the adsorption of Cu(II), Cd(II), Pb(II), CBZ, and TC were carried out using 0.1 mol/L of KH_2PO_4 solution and trichloromethane, respectively. After agitation at 100 rpm for 24 h at T 298 K, HBCs were gathered by centrifugation, then were thoroughly washed three times using DI water and dried at T 333 K overnight.

2. Data Analysis

The kinetic data were fitted by the pseudo-first-order model ($\ln(q_e–q_t) = \ln q_e–k_1t$) and pseudo-second-order model ($t/q_t = 1/(k_2q_e^2)+t/q_e$), where q_t (mg/g) was the sorption capacity at time t (min), and k_1 (L/min) and k_2 (g/(mg min)) were the sorption rate constants. Sorption isotherms were analyzed by the Langmuir model ($q_e = q_{max}K_LC_e/(1+K_LC_e)$) and Freundlich model ($q_e = K_FC_e^{1/n}$), where K_L (L/mg) was the Langmuir sorption coefficient, q_{max} (mg/g) was the maximum sorption capacity, and K_F (mg^{1-n} L^n/g) and n were the sorption and nonlinear coefficients for the Freundlich model, respectively. The thermodynamic parameters, including the standard Gibbs free energy change (ΔG^o), enthalpy change (ΔH^o), and entropy change (ΔS^o), were calculated from $K_d = (C_0–C_e)V/C_em$, $\Delta G° = −RT\ln K°$, $\ln K^o = \Delta So/R–\Delta H^o/(RT)$, and $\Delta G^o = \Delta H^o–T\Delta S^o$, where K_d was the equilibrium constant, T was the absolute temperature (K), and R was the universal gas constant 8.314 J/(mol K). $K°$ was the temperature-dependent equilibrium constant of sorption and could be obtained from the intercept of $\ln K_d$ as a function of C_e.

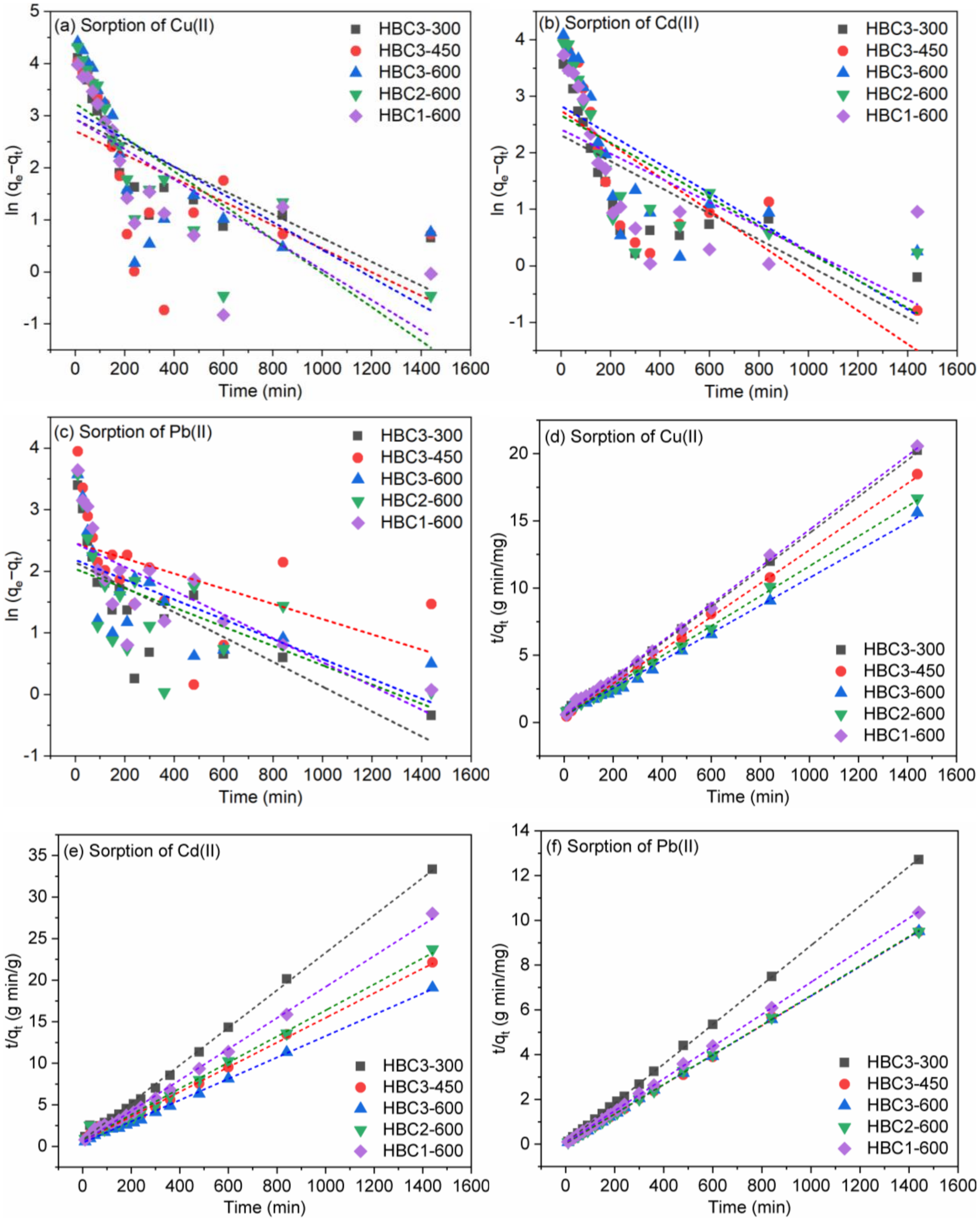

Figure S1. The pseudo-first-order and pseudo-second-order kinetic fittings for the sorption of Cu(II) (**a**,**d**), Cd(II) (**b**,**e**), and Pb(II) (**c**,**f**) on HBCs. $C_{[Cu(II)]initial} = C_{[Cd(II)]initial} = C_{[Pb(II)]initial}$ = 60.0 mg/L, pH = 5.0 ± 0.1, T = 293 K, m/V = 0.5 g/L, I = 0.01 mol/L $NaNO_3$.

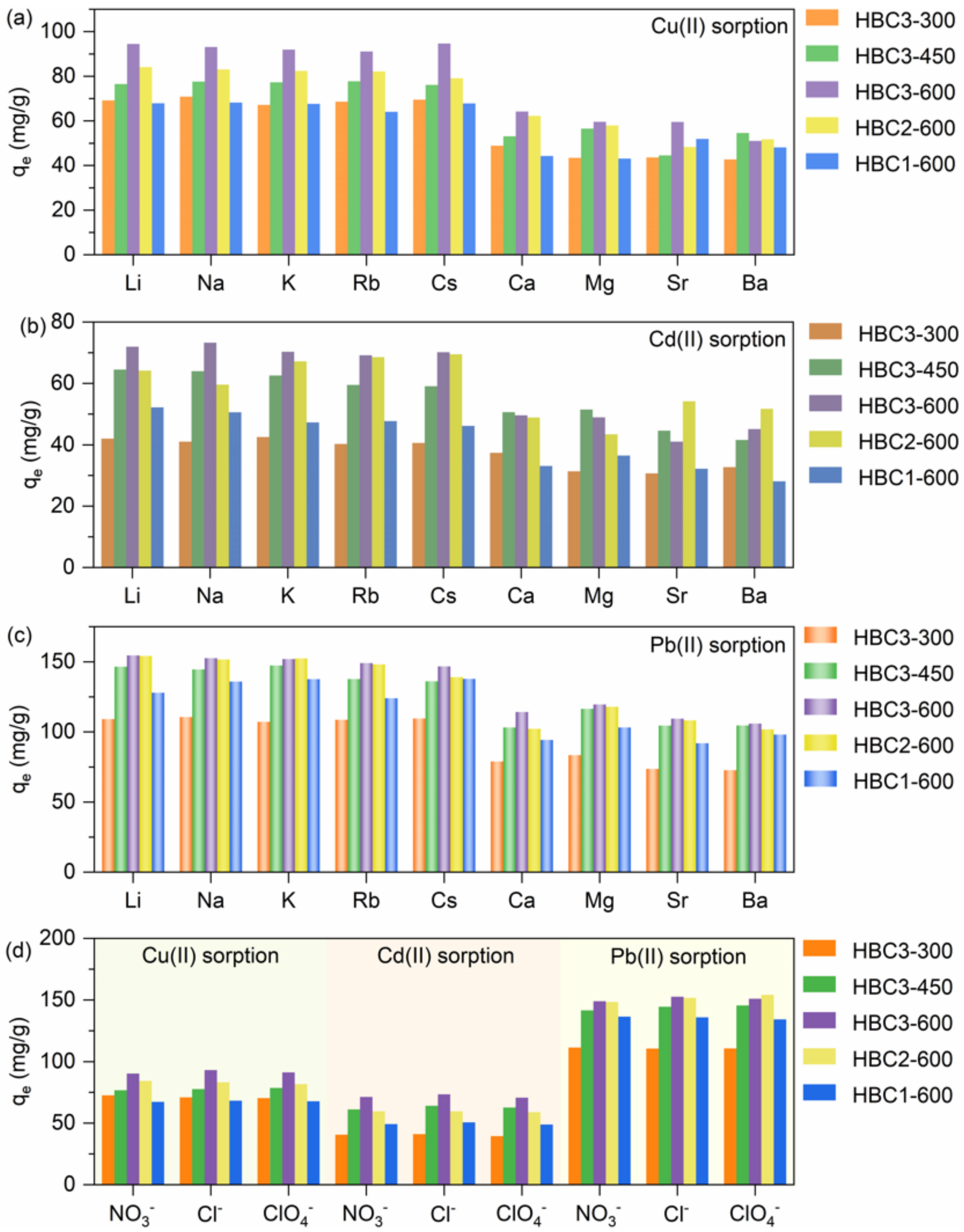

Figure S2. The interference of cations on the adsorption of Cu(II) (**a**), Cd(II) (**b**), and Pb(II) (**c**) on HBCs. The interference of anions (**d**) on the adsorption of Cu(II), Cd(II), and Pb(II) on HBCs. $C_{[background\ X\ ions]}$ = 0.01 mol/L (X=Mg^{2+}, Li^{+}, Rb^{+}, Cs^{+}, Na^{+}, K^{+}, Ca^{2+}, Sr^{2+}, Ba^{2+}), and $C_{[background\ anion]}$ = 0.01 mol/L. $C_{[Cu(II)]initial}$ = $C_{[Cd(II)]initial}$=$C_{[Pb(II)]initial}$ = 60.0 mg/L, pH = 5.0 ± 0.1, T = 293 K, m/V = 0.4 g/L.

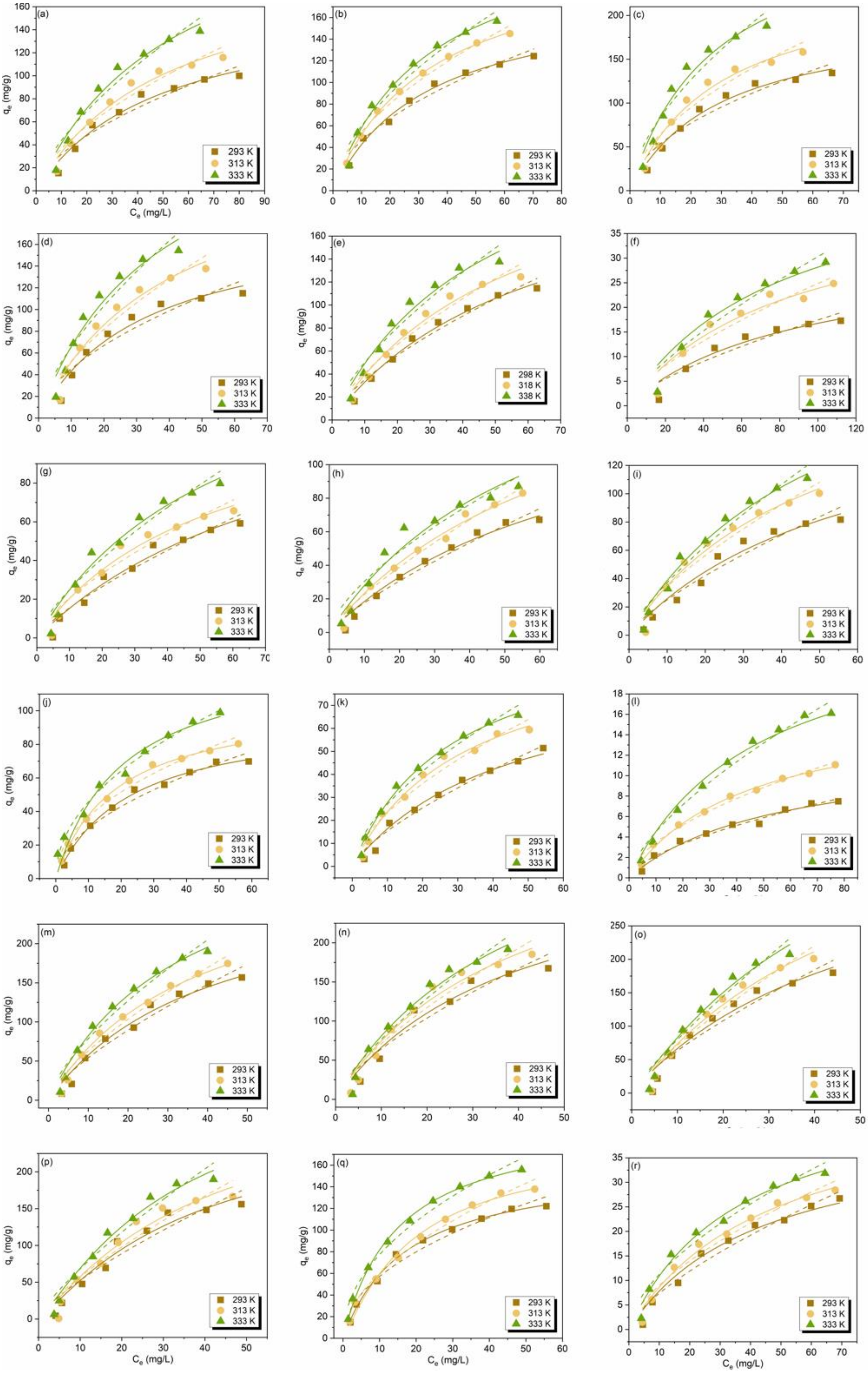
(a)
(b)
(c)
(d)
(e)
(f)
(g)
(h)
(i)
(j)
(k)
(l)
(m)
(n)
(o)
(p)
(q)
(r)
q_e (mg/g)
C_e (mg/L)
293 K
313 K
333 K
298 K
318 K
338 K

Figure S3. Sorption isotherms of Cu(II), Cd(II), and Pb(II) on HBC1-600 (**a**,**g**,**m**), HBC2-600 (**b**,**h**,**n**), HBC3-600 (**c**,**i**,**o**), HBC3-450 (**d**,**j**,**p**), HBC3-300 (**e**,**k**,**q**), and HAP (**f**,**l**,**r**). The solid and dashed lines are the fittings by Langmuir and Freundlich models, respectively. pH = 5.0 ± 0.1, T = 293 K, m/V = 0.50 g/L, I = 0.01 mol/L $NaNO_3$.

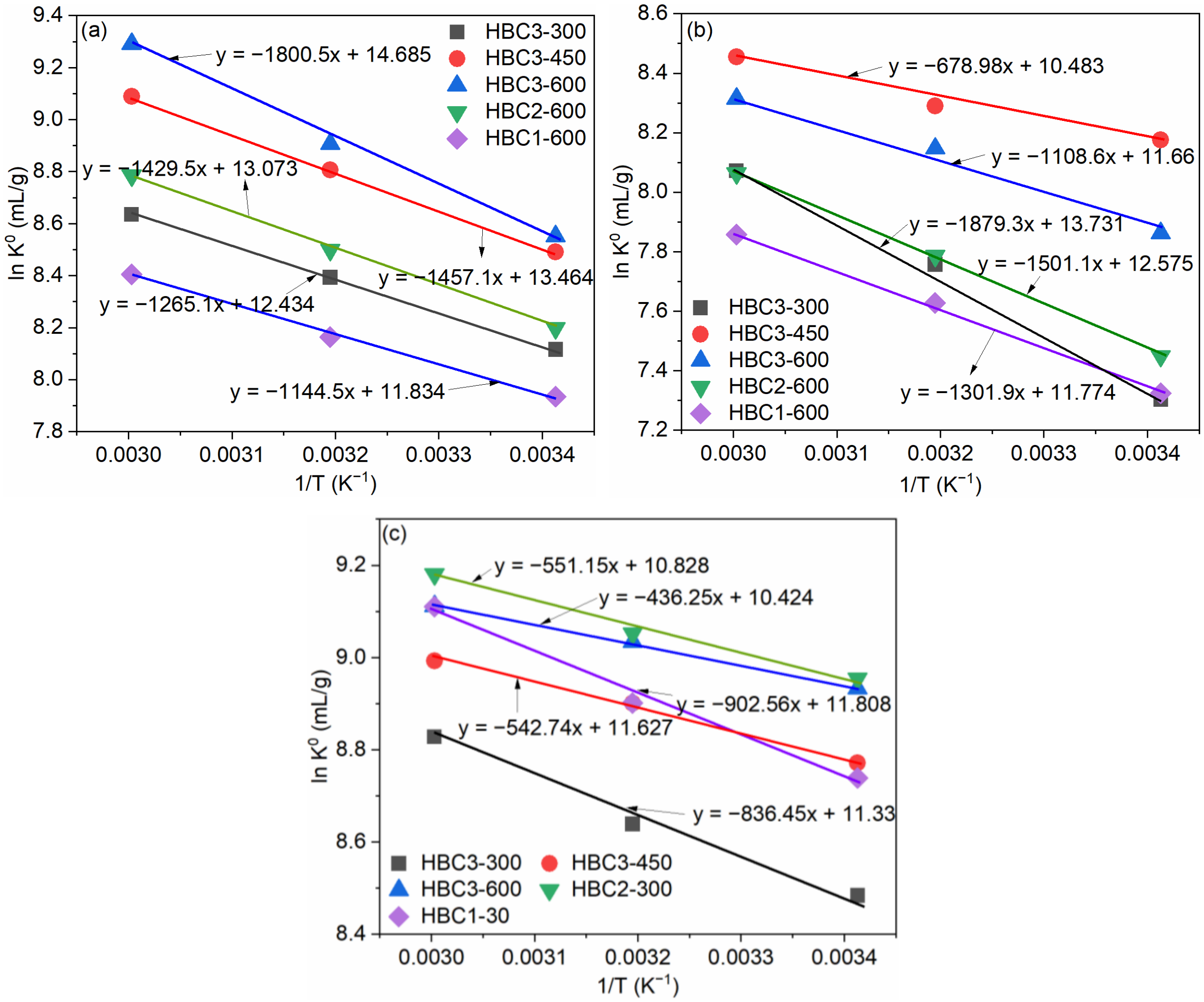

Figure S4. Linear plots of lnK^0 versus 1/T for Cu(II) (**a**), Cd(II) (**b**), and Pb(II) (**c**) adsorbed on HBCs. $C_{[Cu(II)]initial} = C_{[Cd(II)]initial} = C_{[Pb(II)]initial}$=60.0 mg/L, pH = 5.0 ± 0.1, T = 293 K, m/V = 0.5 g/L, I = 0.01 mol/L $NaNO_3$.

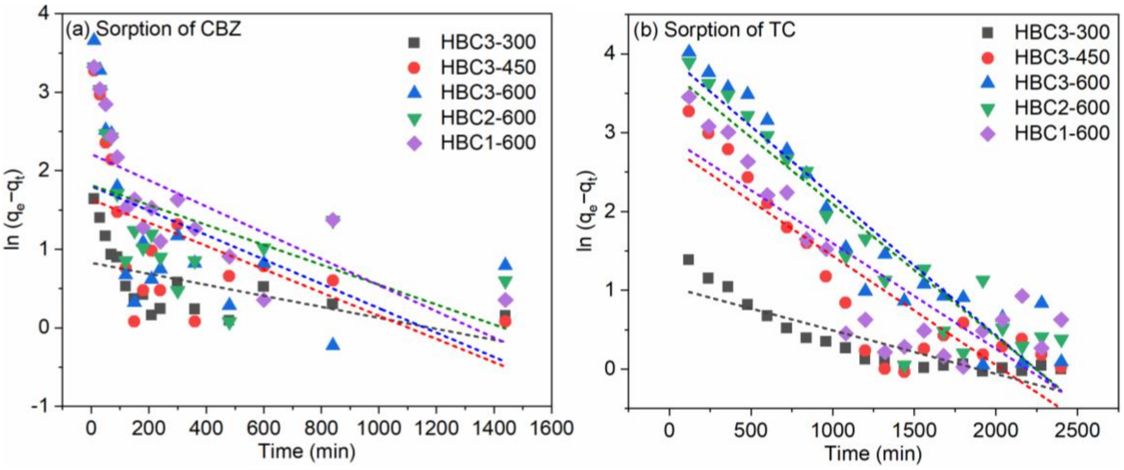

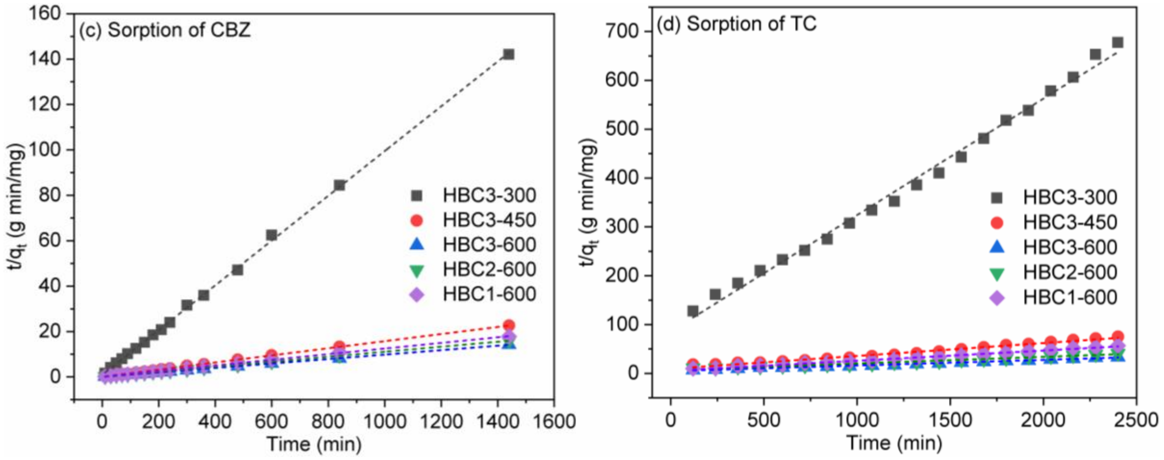

Figure S5. The pseudo-first-order and pseudo-second-order kinetic fittings for the sorption of CBZ (**a**,**c**) and TC (**b**,**d**) on HBCs. $C_{[CBZ]initial}$ = $C_{[TC]initial}$ = 60.0 mg/L, pH = 6.0 ± 0.1, T = 293 K, m/V = 0.5 g/L, I = 0.01 mol/L $NaNO_3$.

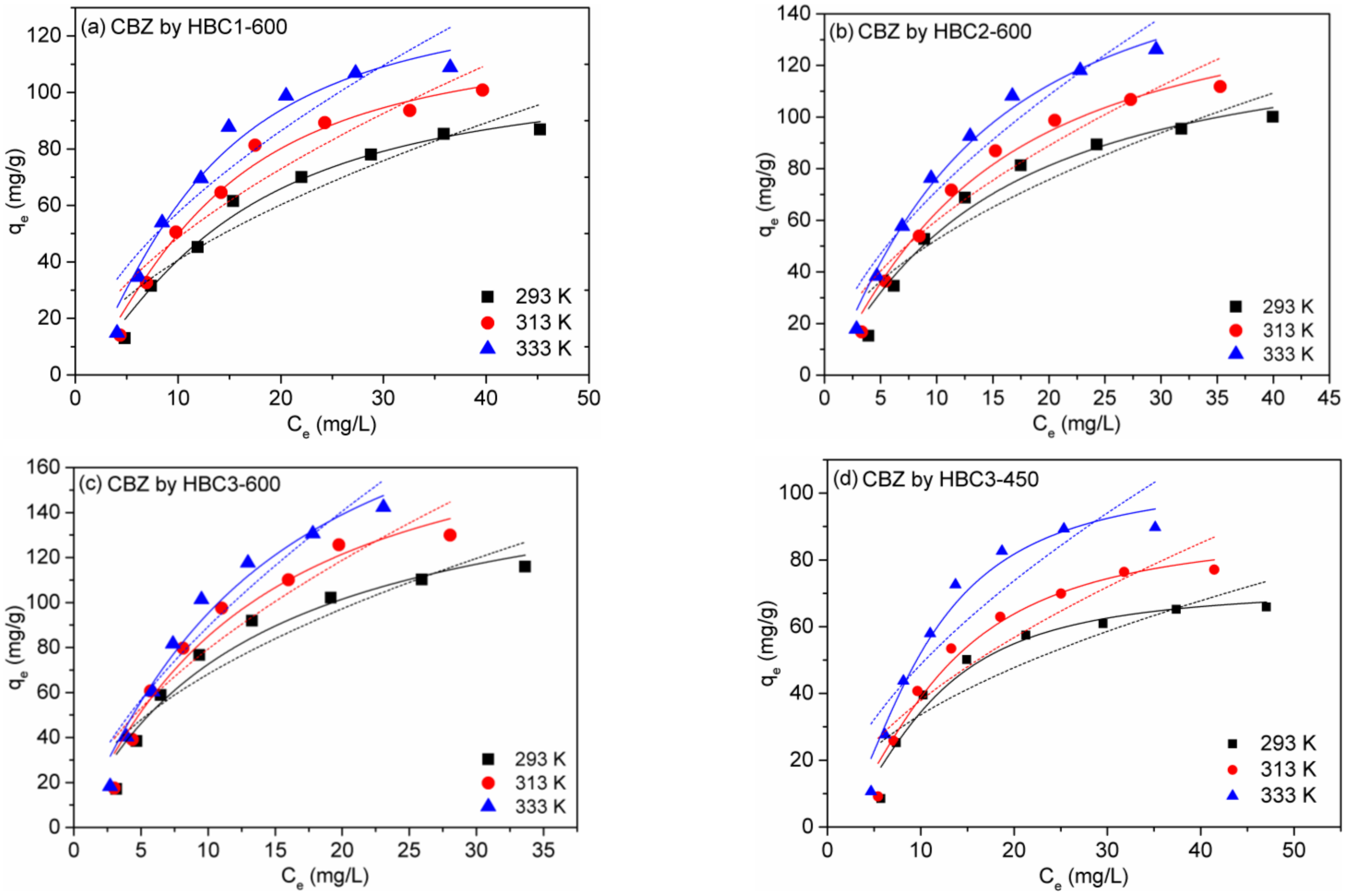

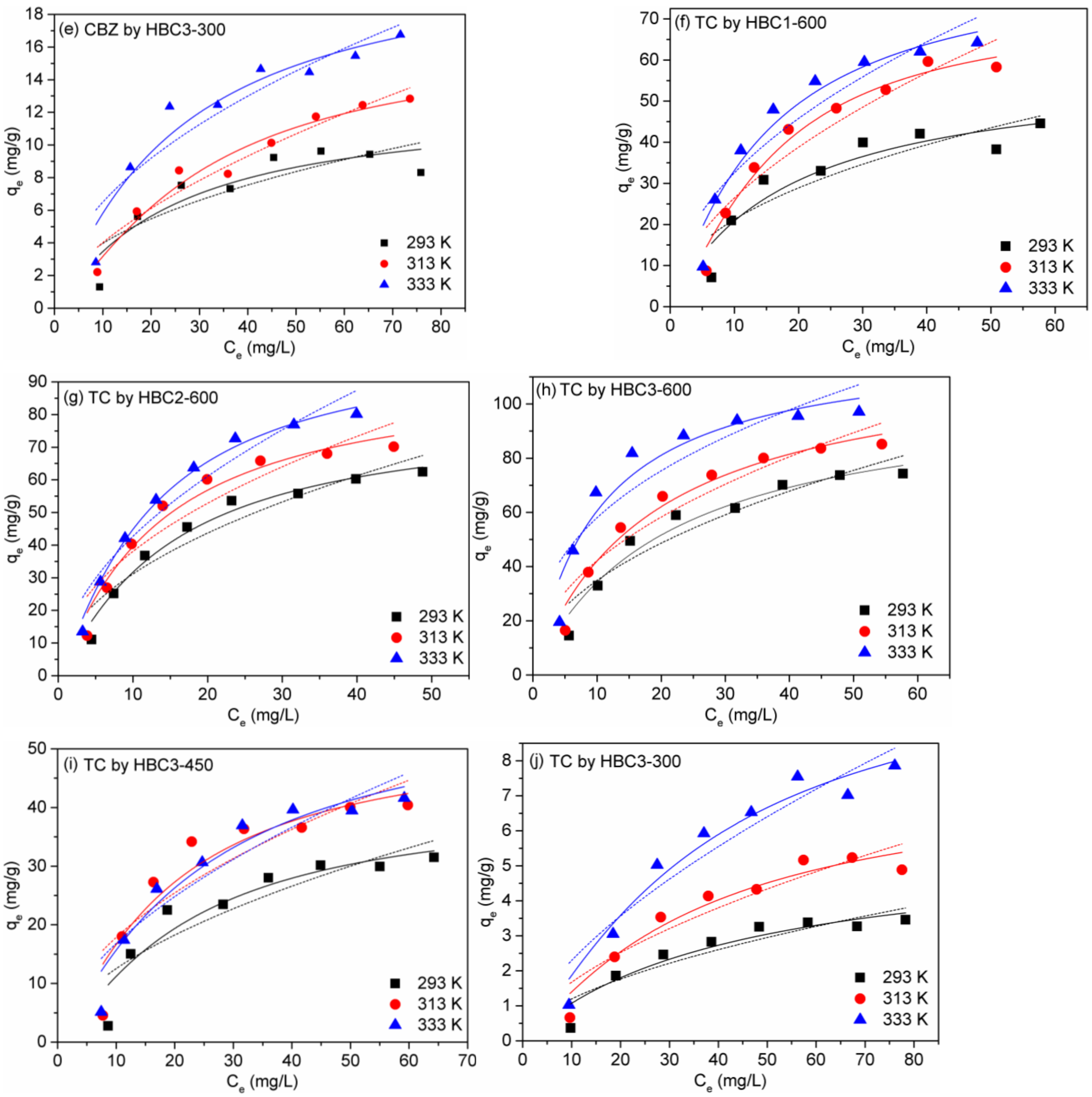

Figure S6. Sorption isotherms of CBZ and TC on HBC1-600 (**a**,**f**), HBC2-600 (**b**,**g**), HBC3-600 (**c**,**h**), HBC3-450 (**d**,**i**), and HBC3-300 (**e**,**j**). The solid and dashed lines are the fittings by Langmuir and Freundlich models, respectively. pH = 6.0 ± 0.1, T = 293 K, m/V = 0.50 g/L, I = 0.01 mol/L $NaNO_3$.

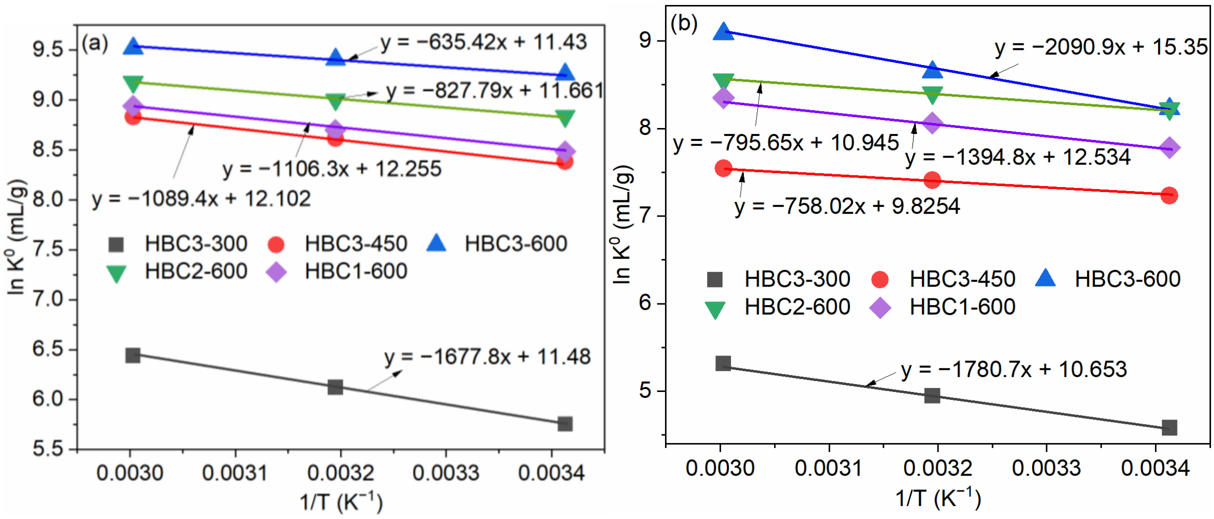

Figure S7. Linear plots of lnK^0 versus 1/T for CBZ (**a**), and TC (**b**) adsorbed on HBCs. $C_{[CBZ]initial}$ = $C_{[TC]initial}$ = 60.0 mg/L, pH = 6.0 ± 0.1, T=293 K, m/V = 0.5 g/L, I = 0.01 mol/L $NaNO_3$.

Table S1. Components of simulated wastewater.

Coexisting Components	Formula Weight	Concentration (mg/L)
$CaCl_2·2H_2O$	147.0	4.0
NaCl	58.4	7.0
NH_4NO_3	80.0	180.0
$K_2HPO_4·3H_2O$	228.2	40.0
CH_3COONa	82.0	205.0
$MgCl_2·6H_2O$	203.3	3.4
C_6H_5COONa	144.1	110.0

Table S2. Deconvolution results of C1s, O1s, and N1s of HBCs.

Elements	Assignment	Binding Energy (eV)	Atom Ratio (%)				
			HBC1-600	HBC2-600	HBC3-600	HBC3-450	HBC3-300
C 1s	C–C	284.8	88.29	81.75	77.32	61.55	57.05
	C–O/sp^2 C–N	286.0	9.41	15.63	18.57	22.35	27.82
	C=O/sp^3 C–N	287.6	2.30	2.62	4.11	9.10	7.97
	O–C=O	289.0	0.00	0.00	0.00	6.99	7.15
O 1s	P=O/C=O	530.9	5.16	13.15	16.98	0.00	0.00
	C–O/P–O	532.9	94.84	86.85	83.02	100.00	100.00
N 1s	pyridinic–N	398.2	32.50	29.80	15.96	16.98	17.26
	amino–N	399.2	20.65	26.30	29.78	23.87	25.79
	pyridonic–N	400.4	36.66	26.84	33.73	39.03	41.96
	quaternary–N	401.7	10.19	17.05	20.54	20.12	14.99

Table S3. Kinetic parameters of the sorption of Cu(II), Cd(II), and Pb(II) on HBCs (T=293 K, pH=5.0±0.1, I=0.01 mol/L $NaNO_3$).

Sorbates	Sorbents	$q_{e,exp}$[a)] (mg/g)	Pseudo-first-order			Pseudo-second-order		
			k_1 (min^{-1})	$q_{e,cal}$[b)] (mg/g)	R^2	k_2 (g/min/mg)	$q_{e,cal}$ (mg/g)	R^2
Cu(II)	HBC3-300	70.9	0.0023	18.8	0.825	0.0004	71.4	0.997
	HBC3-450	77.6	0.0023	15.0	0.745	0.0004	78.1	0.996
	HBC3-600	93.1	0.0027	21.8	0.836	0.0002	94.6	0.995
	HBC2-600	83.1	0.0033	25.4	0.735	0.0003	85.3	0.998
	HBC1-600	68.2	0.0029	18.8	0.664	0.0004	69.9	0.997
Cd(II)	HBC3-300	41.4	0.0023	10.1	0.723	0.0007	42.9	0.997
	HBC3-450	64.3	0.0029	15.5	0.745	0.0004	66.2	0.997
	HBC3-600	73.3	0.0026	17.0	0.642	0.0004	75.3	0.999
	HBC2-600	59.6	0.0024	14.3	0.647	0.0004	61.8	0.999
	HBC1-600	50.6	0.0022	11.2	0.701	0.0007	52.2	0.997
Pb(II)	HBC3-300	110.6	0.0020	8.5	0.687	0.0014	112.1	0.995
	HBC3-450	144.5	0.0012	11.6	0.612	0.0021	147.3	0.999
	HBC3-600	152.6	0.0016	8.9	0.768	0.0015	151.7	0.998
	HBC2-600	151.7	0.0016	7.7	0.757	0.0016	151.3	0.997
	HBC1-600	135.9	0.0019	11.7	0.677	0.0010	137.4	0.996

[a)] Experimental sorption capacity.
[b)] Calculated sorption capacity of pseudo-first or -second-order models.

Table S4. Langmuir and Freundlich fitting parameters for the sorption of Cu(II), Cd(II), and Pb(II) on HBCs.

Sorbents	Sorbates	Temperature (K)	Langmuir parameters			Freundlich parameters		
			K_L (L/mg)	q_{max} (mg/g)	R^2	K_F ($mg^{1-n}L^n/g$)	n	R^2
HBC3-300	Cu(II)	293	0.0160	197.6	0.984	7.179	1.456	0.959
		313	0.0178	239.4	0.973	8.436	1.460	0.942
		333	0.0208	284.5	0.971	10.452	1.470	0.925
	Cd(II)	293	0.0225	88.9	0.986	2.876	1.375	0.971
		313	0.0307	100.8	0.986	5.505	0.596	0.954
		333	0.0347	107.4	0.992	6.573	1.629	0.969
	Pb(II)	293	0.0576	161.8	0.995	18.727	2.066	0.962
		313	0.0359	182.5	0.987	17.290	1.850	0.984
		333	0.0665	204.2	0.997	25.427	2.071	0.976
HBC3-450	Cu(II)	293	0.0303	186.2	0.961	10.330	1.773	0.877
		313	0.0252	256.2	0.971	11.884	1.553	0.909
		333	0.0276	303.8	0.958	14.195	1.510	0.893
	Cd(II)	293	0.0447	97.9	0.993	9.100	1.932	0.964
		313	0.0539	105.8	0.997	12.108	2.068	0.976
		333	0.0489	135.7	0.991	14.838	2.049	0.969
	Pb(II)	293	0.0157	287.5	0.972	9.914	1.321	0.910
		313	0.0159	325.5	0.966	10.672	1.295	0.904
		333	0.0158	356.5	0.979	11.067	1.266	0.939
HBC3-600	Cu(II)	293	0.0334	201.7	0.986	15.768	1.890	0.920
		313	0.0342	246.4	0.979	17.855	1.795	0.918
		333	0.0362	317.8	0.967	21.151	1.675	0.907
	Cd(II)	293	0.0170	176.5	0.984	4.417	1.330	0.936
		313	0.0206	204.3	0.979	6.656	1.403	0.935
		333	0.0188	243.9	0.987	6.449	1.315	0.949

	Pb(II)	293	0.0197	405.7	0.966	10.747	1.298	0.909
		313	0.0158	451.2	0.974	11.016	1.231	0.925
		333	0.0148	525.5	0.977	11.122	1.168	0.930
HBC2-600	Cu(II)	293	0.0297	185.7	0.991	13.319	1.860	0.964
		313	0.0295	226.5	0.998	14.679	1.762	0.977
		333	0.0298	252.7	0.993	15.516	1.707	0.951
	Cd(II)	293	0.0133	156.9	0.982	2.790	1.250	0.959
		313	0.0143	185.9	0.989	3.650	1.267	0.976
		333	0.0165	197.3	0.969	6.610	1.508	0.927
	Pb(II)	293	0.0252	328.5	0.962	13.185	1.452	0.905
		313	0.0250	387.7	0.974	16.569	1.390	0.926
		333	0.0243	413.6	0.976	14.538	1.328	0.938
HBC1-600	Cu(II)	293	0.0202	169.1	0.972	8.136	1.691	0.915
		313	0.0195	204.8	0.977	8.873	1.624	0.924
		333	0.0181	269.0	0.970	9.550	1.512	0.923
	Cd(II)	293	0.0116	128.9	0.984	2.611	1.293	0.945
		313	0.0188	146.8	0.982	4.176	1.444	0.930
		333	0.0173	167.9	0.986	4.714	1.387	0.941
	Pb(II)	293	0.0248	289.9	0.982	10.184	1.379	0.951
		313	0.0266	318.1	0.989	12.183	1.401	0.965
		333	0.0295	360.9	0.988	16.057	1.449	0.957

Table S5. Comparison of q_{max} for the sorption of Cu(II), Cd(II), and Pb(II) on HBCs with other sorbents.

Metals	Sorbents	Experimental Conditions	q_{max} (mg/g)	References
Cu(II)	Amino-modified sawdust biochar	T 293 K, pH 5.0	16.1	[1]
	Alkali-modified hickory biochar	T 298 K, pH 5.0	17.9	[2]
	Pyromellitic dianhydride-rice straw biochar	T 298 K, pH 5.0	24.6	[3]
	MnO_x-hickory wood biochar	T 295 K, pH 6.0	34.2	[4]
	Magnetic marine algae biochar	T 295 K, pH 5.0	46.7	[5]
	Dairy manure biochar	T 298 K, pH 5.0	54.4	[6]
	H_2O_2-treated yak manure biochar	T 298 K, pH 5.0	64.9	[7]
	Multiwalled carbon nanotubes	T 298 K, pH 3.0	118.4	[8]
	Graphene oxide aerogel	T 298 K, pH 6.3	19.7	[9]
	HBC3-600	T 293 K, pH 5.0	201.7	This study
Cd(II)	Zero-valent iron nanoparticle	T 293–298 K, pH 5.0	110	[10]
	Magnetic GO-MgAl-LDH	T 298 K, pH 4.0	45.1	[11]
	Hickory wood biochar	T 295 K, pH 5.0	4.8	[4]
	Ball-milled bamboo biochar	T 298 K, pH 5.45	40.0	[12]
	Hydroxyapatite	T 298 K, pH 5.45	49.4	[13]
	Cauliflower leaves biochar	T 298 K, pH 6.0	73.8	[14]
	Hydrothermal biochar	T 293 K, pH 5.0	128.2	[15]
	Carbon nanotubes	T 303 K, pH 6.0	14.5	[16]
	Graphene oxide	T 298 K, pH 7.0	401.1	[17]
	HBC3-600	T 293 K pH 5.0	176.5	This study
Pb(II)	Hydroxyapatite	T 298 K, pH 5.45	87.5	[13]
	Hickory wood biochar	T 295 K, pH 5.0	71.4	[4]
	Activated carbon	T 298 K, pH 6.0	171.0	[18]
	Magnetic GO-MgAl-LDH	T 298 K, pH 4.0	192.3	[11]

Zero-valent iron nanoparticle	T 293–298K, pH 4.8	170	[10]
Ball-milled wheat straw biochar	T 298 K, pH 5.45	134.7	[19]
Rice straw biochars	T 298 K, pH 6.0	198.2	[20]
Cauliflower leaves biochar	T 298 K, pH 6.0	177.8	[14]
H_2O_2-coconut fiber biochar	T 298 K, pH 6.0	105.5	[21]
Anaerobic digestion sludge biochar	T 298 K, pH 6.0	51.2	[22]
Carbon nanotubes	T 298 K, pH 5.0	49.7	[23]
Graphene oxide	T 288 K, pH 5.0	327.9	[24]
HBC3-600	T 293 K pH 5.0	405.7	This study

Table S6. Thermodynamic parameters for the sorption of Cu(II), Cd(II), and Pb(II) on HBCs.

Sorbents	Metals	T (K)	*ΔH*° (kJ/mol)	*ΔS*° (J/mol K)	*ΔG*° (kJ/mol)
HBC3-300	Cu(II)	293	10.52	103.38	−19.77
		313	10.72		−21.63
		333	10.52		−23.91
	Cd(II)	293	15.66	114.16	−17.79
		313	15.29		−20.45
		333	15.67		−22.35
	Pb(II)	293	6.93	94.19	−20.67
		313	7.16		−22.32
		333	6.93		−24.44
HBC3-450	Cu(II)	293	12.11	111.94	−20.69
		313	12.38		−22.66
		333	12.11		−25.16
	Cd(II)	293	5.62	87.16	−19.92
		313	5.70		−21.57
		333	5.61		−23.41
	Pb(II)	293	4.52	88.35	−21.39
		313	4.49		−23.16
		333	4.52		−24.90
HBC3-600	Cu(II)	293	14.94	122.09	−20.83
		313	15.24		−22.97
		333	14.93		−25.73
	Cd(II)	293	9.25	98.94	−19.15
		313	8.88		−21.46
		333	9.26		−23.02
	Pb(II)	293	3.63	86.67	−21.76
		313	3.46		−23.66
		333	3.93		−25.23
HBC2-600	Cu(II)	293	11.88	108.69	−19.97
		313	11.64		−22.38
		333	11.87		−24.32
	Cd(II)	293	12.49	104.55	−18.15
		313	12.46		−20.26
		333	12.49		−22.33
	Pb(II)	293	4.57	90.03	−21.81
		313	4.70		−23.48
		333	4.56		−25.42
HBC1-600	Cu(II)	293	9.49	98.39	−19.33
		313	9.63		−21.17

	333	9.49		−23.27
Cd(II)	293	10.84	97.89	−17.84
	313	10.79		−19.85
	333	10.84		−21.75
Pb(II)	293	7.62	98.17	−21.29
	313	7.82		−23.06
	333	7.63		−25.22

Table S7. Kinetic parameters of the sorption of CBZ and TC on HBCs (T = 293 K, pH = 6.0 ± 0.1, I = 0.01 mol/L $NaNO_3$).

Sorbates	Sorbents	$q_{e,exp}$ [a)] (mg/g)	Pseudo-first-order			Pseudo-second-order		
			k_1 (min^{-1})	$q_{e,cal}$ [b)] (mg/g)	R^2	k_2 (g/min/mg)	$q_{e,cal}$ (mg/g)	R^2
CBZ	HBC3-300	9.6	0.0007	2.3	0.325	0.0113	9.8	0.998
	HBC3-450	60.9	0.0015	5.1	0.245	0.0024	63.9	0.999
	HBC3-600	102.1	0.0016	6.1	0.218	0.0028	102.0	0.996
	HBC2-600	89.3	0.0013	6.2	0.205	0.0030	90.5	0.998
	HBC1-600	78.1	0.0017	9.1	0.468	0.0014	80.6	0.996
TC	HBC3-300	3.4	0.0006	2.8	0.763	0.0009	3.5	0.994
	HBC3-450	30.2	0.0014	16.8	0.715	0.0002	34.8	0.988
	HBC3-600	70.1	0.0018	52.7	0.742	0.0001	72.4	0.992
	HBC2-600	55.7	0.0017	44.0	0.747	0.0001	57.5	0.991
	HBC1-600	42.1	0.0013	18.9	0.701	0.0002	44.0	0.993

[a)] Experimental sorption capacity.
[b)] Calculated sorption capacity of pseudo-first or -second-order models.

Table S8. Langmuir and Freundlich fitting parameters for the sorption of CBZ and TC on HBCs.

Sorbents	Sorbates	Temperature	Langmuir parameters			Freundlich parameters		
			K_L (L/mg)	q_{max} (mg/g)	R^2	K_F ($mg^{1-n}L^n/g$)	n	R^2
HBC3-300	CBZ	293	0.031	12.5	0.924	1.367	2.159	0.692
		313	0.011	16.5	0.972	0.991	1.645	0.934
		333	0.029	21.9	0.939	2.054	1.996	0.846
	TC	293	0.023	5.7	0.904	0.329	1.783	0.823
		313	0.015	7.6	0.953	0.437	1.689	0.857
		333	0.012	11.3	0.966	0.528	1.638	0.892
HBC3-450	CBZ	293	0.014	71.7	0.973	10.51	1.980	0.797
		313	0.015	89.2	0.983	9.91	1.715	0.832
		333	0.014	104.8	0.986	12.27	1.669	0.799
	TC	293	0.021	41.1	0.913	3.59	1.845	0.778
		313	0.028	52.9	0.904	5.49	1.953	0.763
		333	0.026	59.3	0.945	4.62	1.783	0.843
HBC3-600	CBZ	293	0.075	168.9	0.965	21.01	1.957	0.873
		313	0.061	191.1	0.968	20.73	1.715	0.883
		333	0.052	223.8	0.976	19.79	1.529	0.912
	TC	293	0.048	105.1	0.963	11.57	2.088	0.886
		313	0.055	118.5	0.973	14.39	2.141	0.891
		333	0.087	132.7	0.931	21.37	2.653	0.792
HBC2-600	CBZ	293	0.049	134.8	0.984	15.51	1.890	0.884
		313	0.044	151.5	0.983	15.15	1.757	0.912

		333	0.047	174.7	0.988	17.83	1.658	0.927
	TC	293	0.048	80.1	0.984	10.16	2.053	0.896
		313	0.059	92.4	0.971	12.83	2.114	0.966
		333	0.051	103.1	0.991	12.98	1.934	0.924
HBC1-600	CBZ	293	0.026	108.8	0.983	11.06	1.761	00899
		313	0.023	120.9	0.981	12.52	1.701	0.888
		333	0.027	136.8	0.971	14.87	1.704	0.859
	TC	293	0.055	58.6	0.911	7.58	2.237	0.772
		313	0.023	72.8	0.981	7.42	1.812	0.883
		333	0.048	84.9	0.948	10.47	2.033	0.848

Table S9. Comparison of q_{max} for the sorption of CBZ and TC on HBCs with other sorbents.

Metals	Sorbents	Experimental conditions	q_{max} (mg/g)	References
CBZ	Biochar/Fe_3O_4	pH 7.0	62.7	[25]
	Activated carbon/Fe_3O_4	pH 7.0	135.1	[25]
	Zirconium MOF UiO-66	pH 6.0	37.2	[26]
	Activated palm kernel shell	pH 7.0	62.4	[27]
	MOF-derived magnetic porous carbon	pH 7.2	37.9	[28]
	SWCNT	pH 7.0	185	[29]
	Pine-wood nanobiochar	pH 6.0	116	[30]
	MWCNT-COOH	pH 7.2	110	[31]
	Graphene oxide	pH 7.2	215	[31]
	HBC3-600	pH 6.0	168.9	This study
TC	Biochar/Fe_3O_4	pH 7.0	94.2	[25]
	Activated carbon/Fe_3O_4	pH 7.0	45.3	[25]
	Zirconium MOF UiO-66	pH 6.0	23.1	[26]
	Sewage sludge biochar	pH 7.0	5.8	[32]
	Activated sludge-biochar	pH 7.0	116.9	[33]
	Pharmaceutical sludge biochar	pH 6.0	157.4	[34]
	Ferric-activated sludge sorbent	pH 6.0	40.8	[35]
	Iron-loaded sludge biochar	pH 2.0–10.0	104.9	[36]
	Fe/S wasted sludge biochar	pH 3.0	174.1	[37]
	HBC3-600	pH 6.0	105.1	This study

Table S10. Thermodynamic parameters for the sorption of CBZ and TC on HBCs.

Sorbents	Pharmaceuticals	T (K)	$\Delta H°$ (kJ/mol)	$\Delta S°$ (J/mol K)	$\Delta G°$ (kJ/mol)
HBC3-300	CBZ	293	13.95	95.44	−14.01
		313	14.49		−15.37
		333	13.95		−17.83
	TC	293	14.79	88.57	−11.16
		313	14.75		−12.87
		333	14.78		−14.71
HBC3-450	CBZ	293	9.05	100.62	−20.43
		313	9.33		−22.16
		333	9.05		−24.46
	TC	293	6.15	81.69	−17.62
		313	6.12		−19.29

		333	6.14		−20.89
HBC3-600	CBZ	293	5.29	95.03	−22.55
		313	5.26		−24.49
		333	5.30		−26.35
	TC	293	17.36	127.62	−20.04
		313	17.84		−22.11
		333	17.34		−25.15
HBC2-600	CBZ	293	6.87	96.95	−21.53
		313	7.04		−23.31
		333	6.87		−25.42
	TC	293	6.49	90.99	−20.04
		313	6.33		−22.01
		333	6.47		−23.68
HBC1-600	CBZ	293	9.18	101.89	−20.68
		313	9.25		−22.64
		333	9.17		−24.76
	TC	293	11.58	104.21	−18.95
		313	11.77		−20.85
		333	11.57		−23.13

References

1. Yang, G.-X.; Jiang, H. Amino modification of biochar for enhanced adsorption of copper ions from synthetic wastewater. *Water Res.* **2014**, *48*, 396–405, https://doi.org/10.1016/j.watres.2013.09.050.

2. Ding, Z.; Hu, X.; Wan, Y.; Wang, S.; Gao, B. Removal of lead, copper, cadmium, zinc, and nickel from aqueous solutions by alkali-modified biochar: Batch and column tests. *J. Ind. Eng. Chem.* **2016**, *33*, 239–245, https://doi.org/10.1016/j.jiec.2015.10.007.

3. Deng, J.; Liu, Y.; Liu, S.; Zeng, G.; Tan, X.; Huang, B.; Tang, X.; Wang, S.; Hua, Q.; Yan, Z. Competitive adsorption of Pb(II), Cd(II) and Cu(II) onto chitosan-pyromellitic dianhydride modified biochar. *J. Colloid Interface Sci.* **2017**, *506*, 355–364, https://doi.org/10.1016/j.jcis.2017.07.069.

4. Wang, H.; Gao, B.; Wang, S.; Fang, J.; Xue, Y.; Yang, K. Removal of Pb(II), Cu(II), and Cd(II) from aqueous solutions by biochar derived from KMnO4 treated hickory wood. *Bioresour. Technol.* **2015**, *197*, 356–362, https://doi.org/10.1016/j.biortech.2015.08.132.

5. Son, E.-B.; Poo, K.-M.; Chang, J.-S.; Chae, K.-J. Heavy metal removal from aqueous solutions using engineered magnetic biochars derived from waste marine macro-algal biomass. *Sci. Total Environ.* **2018**, *615*, 161–168, https://doi.org/10.1016/j.scitotenv.2017.09.171.

6. Xu, X.; Cao, X.; Zhao, L.; Wang, H.; Yu, H.; Gao, B. Removal of Cu, Zn, and Cd from aqueous solutions by the dairy manure-derived biochar. *Environ. Sci. Pollut. Res.* **2012**, *20*, 358–368, https://doi.org/10.1007/s11356-012-0873-5.

7. Wang, Y.; Liu, R. H2O2 treatment enhanced the heavy metals removal by manure biochar in aqueous solutions. *Sci. Total Environ.* **2018**, *628-629*, 1139–1148, https://doi.org/10.1016/j.scitotenv.2018.02.137.

8. Gupta, V.K.; Agarwal, S.; Bharti, A.K.; Sadegh, H. Adsorption mechanism of functionalized multi-walled carbon nanotubes for advanced Cu (II) removal. *J. Mol. Liq.* **2017**, *230*, 667–673, https://doi.org/10.1016/j.molliq.2017.01.083.

9. Mi, X.; Huang, G.; Xie, W.; Wang, W.; Liu, Y.; Gao, J. Preparation of graphene oxide aerogel and its adsorption for Cu2+ ions. *Carbon* **2012**, *50*, 4856–4864, https://doi.org/10.1016/j.carbon.2012.06.013.

10. Yoon, K.; Cho, D.-W.; Tsang, D.; Bolan, N.; Rinklebe, J.; Song, H. Fabrication of engineered biochar from paper mill sludge and its application into removal of arsenic and cadmium in acidic water. *Bioresour. Technol.* **2017**, *246*, 69–75, https://doi.org/10.1016/j.biortech.2017.07.020.

11. Huang, Q., Chen, Y., Yu, H., Yan, L., Zhang, J., Wang, B., Du, B., Xing, L. Magnetic graphene oxide/MgAl-layered double hydroxide nanocomposite: one-pot solvothermal synthesis, adsorption performance and mechanisms for Pb2+, Cd2+, and Cu2+. *Chem. Eng. J.* **2018**, 341, 1–9, https://doi.org/10.1016/j.cej.2018.01.156.

12. Wang, B.; Gao, B.; Wan, Y. Entrapment of ball-milled biochar in Ca-alginate beads for the removal of aqueous Cd(II). *J. Ind. Eng. Chem.* **2017**, *61*, 161–168, https://doi.org/10.1016/j.jiec.2017.12.013.

13. Chen, S.B.; Ma, Y.B.; Chen, L.; Xian, K. Adsorption of aqueous Cd2+, Pb2+, Cu2+ ions by nano-hydroxyapatite: Single- and multi-metal competitive adsorption study. *Geochem. J.* **2010**, *44*, 233–239, https://doi.org/10.2343/geochemj.1.0065.

14. Ahmad, Z.; Gao, B.; Mosa, A.; Yu, H.; Yin, X.; Bashir, A.; Ghoveisi, H.; Wang, S. Removal of Cu(II), Cd(II) and Pb(II) ions from aqueous solutions by biochars derived from potassium-rich biomass. *J. Clean. Prod.* **2018**, *180*, 437–449, https://doi.org/10.1016/j.jclepro.2018.01.133.

15. Zhao, X.; Li, M.; Zhai, F.; Hou, Y.; Hu, R. Phosphate modified hydrochars produced via phytic acid-assisted hydrothermal carbonization for efficient removal of U(VI), Pb(II) and Cd(II). *J. Environ. Manag.* **2021**, *298*, 113487, https://doi.org/10.1016/j.jenvman.2021.113487.

16. Sun, W.; Jiang, B.; Wang, F.; Xu, N. Effect of carbon nanotubes on Cd(II) adsorption by sediments. *Chem. Eng. J.* **2014**, *264*, 645–653, https://doi.org/10.1016/j.cej.2014.11.137.

17. Guo, T.; Bulin, C.; Ma, Z.; Li, B.; Zhang, Y.; Zhang, B.; Xing, R.; Ge, X. Mechanism of Cd(II) and Cu(II) Adsorption onto Few-Layered Magnetic Graphene Oxide as an Efficient Adsorbent. *ACS Omega* **2021**, *6*, 16535–16545, https://doi.org/10.1021/acsomega.1c01770.

18. Kyzas, G.Z.; Bomis, G.; Kosheleva, R.I.; Efthimiadou, E.K.; Favvas, E.; Kostoglou, M.; Mitropoulos, A.C. Nanobubbles effect on heavy metal ions adsorption by activated carbon. *Chem. Eng. J.* **2018**, *356*, 91–97, https://doi.org/10.1016/j.cej.2018.09.019.

19. Cao, Y.; Xiao, W.; Shen, G.; Ji, G.; Zhang, Y.; Gao, C.; Han, L. Carbonization and ball milling on the enhancement of Pb(II) adsorption by wheat straw: Competitive effects of ion exchange and precipitation. *Bioresour. Technol.* **2018**, *273*, 70–76, https://doi.org/10.1016/j.biortech.2018.10.065.

20. Shen, Z.; Hou, D.; Jin, F.; Shi, J.; Fan, X.; Tsang, D.; Alessi, D. Effect of production temperature on lead removal mechanisms by rice straw biochars. *Sci. Total Environ.* **2018**, *655*, 751–758, https://doi.org/10.1016/j.scitotenv.2018.11.282.

21. Wu, W.; Li, J.; Lan, T.; Mueller, K.; Niazi, N.K.; Chen, X.; Xu, S.; Zheng, L.; Chu, Y.; Li, J.; et al. Unraveling sorption of lead in aqueous solutions by chemically modified biochar derived from coconut fiber: A microscopic and spectroscopic investigation. *Sci. Total Environ.* **2016**, *576*, 766–774, https://doi.org/10.1016/j.scitotenv.2016.10.163.

22. Ho, S.-H.; Chen, Y.-D.; Yang, Z.-K.; Nagarajan, D.; Chang, J.-S.; Ren, N.-Q. High-efficiency removal of lead from wastewater by biochar derived from anaerobic digestion sludge. *Bioresour. Technol.* **2017**, *246*, 142–149, https://doi.org/10.1016/j.biortech.2017.08.025.

23. Wang, H.; Zhou, A.; Peng, F.; Yu, H.; Chen, L. Adsorption characteristic of acidified carbon nanotubes for heavy metal Pb(II) in aqueous solution. *Mater. Sci. Eng. A* **2007**, *466*, 201–206, https://doi.org/10.1016/j.msea.2007.02.097.

24. Bai, C.; Wang, L.; Zhu, Z. Adsorption of Cr(III) and Pb(II) by graphene oxide/alginate hydrogel membrane: Characterization, adsorption kinetics, isotherm and thermodynamics studies. *Int. J. Biol. Macromol.* **2019**, *147*, 898–910, https://doi.org/10.1016/j.ijbiomac.2019.09.249.

25. Shan, D.; Deng, S.; Zhao, T.; Wang, B.; Wang, Y.; Huang, J.; Yu, G.; Winglee, J.; Wiesner, M.R. Preparation of ultrafine magnetic biochar and activated carbon for pharmaceutical adsorption and subsequent degradation by ball milling. *J. Hazard. Mater.* **2015**, *305*, 156–163, https://doi.org/10.1016/j.jhazmat.2015.11.047.

26. Chen, C.; Chen, D.; Xie, S.; Quan, H.; Luo, X.; Guo, L. Adsorption Behaviors of Organic Micropollutants on Zirconium Metal–Organic Framework UiO-66: Analysis of Surface Interactions. *ACS Appl. Mater. Interfaces* **2017**, *9*, 41043–41054, https://doi.org/10.1021/acsami.7b13443.

27. To, M.-H.; Hadi, P.; Hui, C.-W.; Lin, C.S.K.; McKay, G. Mechanistic study of atenolol, acebutolol and carbamazepine adsorption on waste biomass derived activated carbon. *J. Mol. Liq.* **2017**, *241*, 386–398, https://doi.org/10.1016/j.molliq.2017.05.037.

28. Chen, D.; Chen, C.; Shen, W.; Quan, H.; Chen, S.; Xie, S.; Luo, X.; Guo, L. MOF-derived magnetic porous carbon-based sorbent: Synthesis, characterization, and adsorption behavior of organic micropollutants. *Adv. Powder Technol.* **2017**, *28*, 1769–1779, https://doi.org/10.1016/j.apt.2017.04.018.
29. Liu, F.-F.; Zhao, J.; Wang, S.; Du, P.; Xing, B. Effects of Solution Chemistry on Adsorption of Selected Pharmaceuticals and Personal Care Products (PPCPs) by Graphenes and Carbon Nanotubes. *Environ. Sci. Technol.* **2014**, *48*, 13197–13206, https://doi.org/10.1021/es5034684.
30. Naghdi, M.; Taheran, M.; Pulicharla, R.; Rouissi, T.; Brar, S.K.; Verma, M.; Surampalli, R. Pine-wood derived nanobiochar for removal of carbamazepine from aqueous media: Adsorption behavior and influential parameters. *Arab. J. Chem.* **2019**, *12*, 5292–5301, https://doi.org/10.1016/j.arabjc.2016.12.025.
31. Cai, N.; Larese-Casanova, P. Sorption of carbamazepine by commercial graphene oxides: A comparative study with granular activated carbon and multiwalled carbon nanotubes. *J. Colloid Interface Sci.* **2014**, *426*, 152–161, https://doi.org/10.1016/j.jcis.2014.03.038.
32. Zhao, J.; Gao, F.; Sun, Y.; Fang, W.; Li, X.; Dai, Y. New use for biochar derived from bovine manure for tetracycline removal. *J. Environ. Chem. Eng.* **2021**, *9*, 105585, https://doi.org/10.1016/j.jece.2021.105585.
33. Chen, W.; Zhao, B.; Guo, Y.; Guo, Y.; Zheng, Z.; Pak, T.; Li, G. Effect of hydrothermal pretreatment on pyrolyzed sludge biochars for tetracycline adsorption. *J. Environ. Chem. Eng.* **2021**, *9*, 106557, https://doi.org/10.1016/j.jece.2021.106557.
34. Liu, H.; Xu, G.; Li, G. The characteristics of pharmaceutical sludge-derived biochar and its application for the adsorption of tetracycline. *Sci. Total Environ.* **2020**, *747*, 141492, https://doi.org/10.1016/j.scitotenv.2020.141492.
35. Yang, X.; Xu, G.; Yu, H.; Zhang, Z. Preparation of ferric-activated sludge-based adsorbent from biological sludge for tetracycline removal. *Bioresour. Technol.* **2016**, *211*, 566–573, https://doi.org/10.1016/j.biortech.2016.03.140.
36. Wei, J.; Liu, Y.; Li, J.; Zhu, Y.; Yu, H.; Peng, Y. Adsorption and co-adsorption of tetracycline and doxycycline by one-step synthesized iron loaded sludge biochar. *Chemosphere* **2019**, *236*, 124254, https://doi.org/10.1016/j.chemosphere.2019.06.224.
37. Rossmo, K.; Harries, K. The geospatial structure of terrorist cells. *Justice Q.* **2011**, *28*, 221–248. https://doi.org/10.1080/07418820903426197.
